# Isolated Fibula Fracture With Development of Acute Compartment Syndrome

**DOI:** 10.5435/JAAOSGlobal-D-24-00100

**Published:** 2025-03-11

**Authors:** Jeffery M. Bortman, Michael W. Buchanan, David M. Freccero

**Affiliations:** From the Department of Orthopaedic Surgery, Boston Medical Center Pl, Boston, MA.

## Abstract

Compartment syndrome is an orthopaedic emergency with moderate-to-severe sequela (pain, muscle contracture, nerve damage, infection, rhabdomyolysis, renal failure, etc.) if inadequately treated and can be difficult to diagnose in a timely fashion. Further complicating timely diagnosis are atypical presentations resulting in compartment syndrome. This case concerns a 31-year-old man who presented with isolated left closed transverse fibular shaft fracture after being a pedestrian struck by a high-speed motor vehicle. He was not on any anticoagulation at the time of the accident. Initial examination 1 hour after arrival was concerning for compartment syndrome because of firm lower extremity compartments, and intracompartmental pressures met criteria for the diagnosis of acute compartment syndrome. He was emergently taken for dual-incision, four-compartment fasciotomy 3 hours after arrival. This case report demonstrates a presentation of acute compartment syndrome in the absence of tibia fracture or risk factors such as anticoagulation. Clinical suspicion of acute compartment syndrome based on physical examination findings warrants close monitoring and possible compartment release even with atypical presentations such as an isolated fibula fracture, high-energy soft-tissue injuries, or crush injuries.

Acute compartment syndrome is the physiologic cycle of injury leading to inflammation, edema, and hemorrhage within the myofascial envelope, causing increased compartment pressure and subsequent decreased venous output. Decreased circulation leads to ischemia and tissue damage, leading to additional vascular permeability and worsening edema, causing a positive feedback loop resulting in additional increased pressures and rapid progression of tissue ischemia.^1^ In plain language, compartment syndrome is swelling around the muscles leading to increased pressures that can ultimately damage local tissues by restricting blood flow. Classic clinical symptoms of compartment syndrome include the “5 P's”—pain, pallor, paresthesia, pulselessness, and paralysis. Compartment syndrome is frequently associated with fracture; however, older patients and patients with more comorbidities are more likely to have an atypical compartment syndrome in the absence of fracture, often in the setting of high-energy soft-tissue injury or crush injury.^2^ This orthopaedic emergency with moderate-to-severe sequela if inadequately treated can be difficult to diagnose in a timely fashion, with 91% of surveyed trauma surgeons responding that they have seen a missed compartment syndrome during their practice.^3^ Timely diagnosis is complicated by atypical presentations resulting in compartment syndrome. Several studies have demonstrated strong correlation between tibia fractures and compartment syndrome, leading to high suspicion for compartment syndrome after tibia fracture.^4^ However, few studies have demonstrated compartment syndrome after isolated fibula fracture.^5,6^ The tibia is a large, weight-bearing bone with a high density of surrounding musculature, making it particularly prone to increased compartment pressures after injury. By contrast, the fibula is a smaller bone with less surrounding muscle mass, typically with less of an effect on intracompartmental pressures when injured. In this case report, we present a young patient, not on anticoagulation, who was a pedestrian struck with isolated transverse midshaft fibula fracture who subsequently developed compartment syndrome requiring four-compartment fasciotomies.

## Case Report

**Figure 1:**
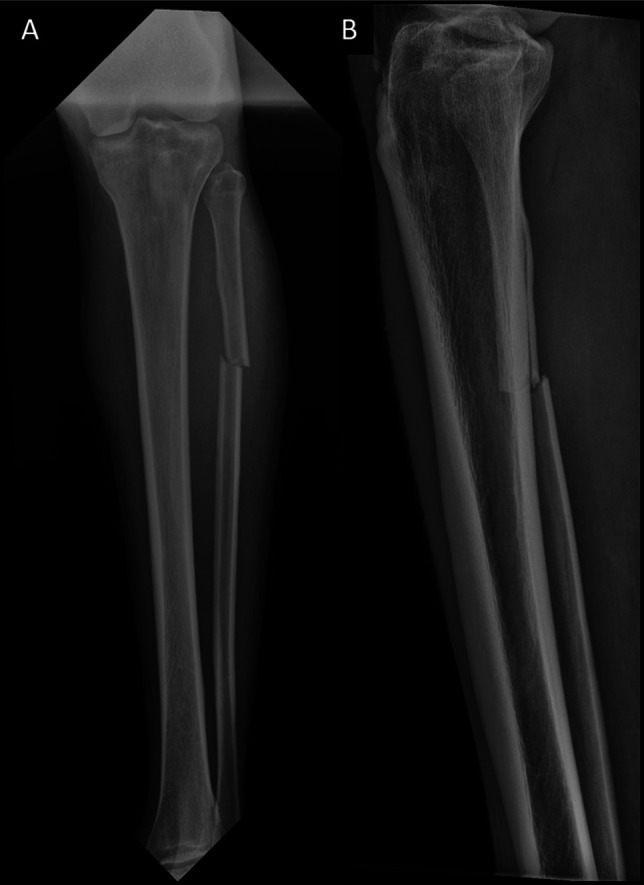
Anteroposterior and lateral radiographs of the left tibia and fibula demonstrating isolated fibular shaft fracture on the day of presentation. Fracture is less than 50% displaced in both planes although it is located in an area of higher soft-tissue volume.

The patient was a 31-year-old man with a medical history notable for depression, polysubstance use disorder, overdose-related cardiac arrest requiring implantable cardiac defibrillator placement, and left eye blindness who presented with isolated left closed transverse fibular shaft fracture (Figure 1), right closed scapular body fracture, and right medial knee soft-tissue laceration without knee joint involvement after being a pedestrian struck. Per report, the patient was struck by a car at approximately 20 miles per hour. However, the patient was unable to provide additional information regarding the circumstances or mechanism surrounding the incident because of altered mental status due to intoxication. Associated injuries included posterior right first and second rib fractures, sternal fracture, and pulmonary contusions.

On arrival, the patient underwent initial trauma workup by the trauma and acute care service team at our Level 1 trauma center. Orthopaedic surgery was paged to evaluate the fibula and scapula fractures 66 minutes after arrival. On initial examination, the patient was altered from substance use (oriented to self only) and his left leg was diffusely firm, most markedly about the posterior compartments, though with increased firmness of the anterior and lateral compartments when compared with the contralateral leg. He also had moderate pain during passive ankle and great toe flexion and extension, and some superficial abrasions about the leg—more specifically, the patient would yell out during manipulation of the ankle and leg but could not verbalize his concerns because of his intoxication. As it relates to the “5 P's,” the patient was experiencing pain. However, he had no pallor, paresthesia, pulselessness, or paralysis. Given his concerning clinical examination and altered mental status, the decision was made to conduct intracompartmental pressure monitoring using a Stryker intracompartmental needle-based pressure monitor in all four compartments of the leg. The diastolic pressure at the time of monitoring was 102 mm Hg. Intracompartmental pressures were as follows: anterior 20 mm Hg, lateral 23 mm Hg, superficial posterior 47 mm Hg, and deep posterior 58 mm Hg. Given the elevated absolute posterior compartment pressures (using a definition of absolute compartment pressure >30 mm Hg) and concerning clinical examination, resident concern was expressed to the attending surgeon. On the attending surgeon's evaluation, the patient's swelling was stable, yet the compartments remained firm, and they agreed that the combination pain on examination and elevated posterior compartment pressures indicated this patient for prophylactic fasciotomies to prevent additional possible myonecrosis and made the decision to take the patient for emergent dual-incision, four-compartment fasciotomies.

**Figure 2:**
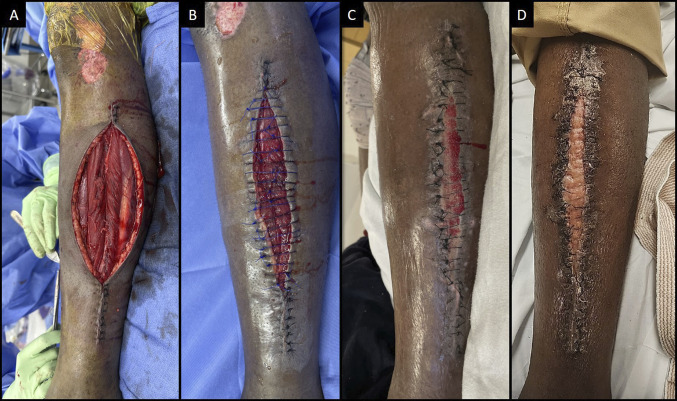
Clinical photographs of the lateral fasciotomy wound on (**A**) the day of presentation after large initial incision to ensure adequate facial release and subsequent partial, tension-free, closure; (**B**) return to OR on POD5 with partial approximation of wound using nonabsorbable suture; (**C**) bedside dressing change with granulation tissue forming and no signs of infection; and (**D**) follow-up in the emergency department on POD22 with epithelization of the wound. OR = operating room; POD = postoperative day.

The patient was taken to the operating room (OR) for release 3 hours after arrival. Intraoperatively, the anterior and lateral compartment muscles herniated through the fasciotomy sites, but the posterior deep and superficial compartments did not (Figure 2, A). All visualized muscles were viable as confirmed with electrocautery. Negative pressure wound therapy was applied to both incisions. The patient subsequently returned to the OR 3 days after the initial fasciotomies for partial closure of the lateral incision, with reapplication of negative pressure therapy (Figure 2, B), and full closure of the medial incision (not pictured). Overall, he returned to the OR 3 times before definitive closure of the lateral incision was achieved, allowing the remaining superficial tissue gap to granulate through secondary intention using a daily regimen of dry sterile dressings (Figure 2, C). The isolated fibula fracture was managed nonsurgically. On the floor, the patient's pain was well controlled with medication, and they maintained the ability to plantarflex and dorsiflex the ankle and toes with near-full strength and maintained full sensation in the superficial peroneal, deep peroneal, and tibial nerve distributions at all time points throughout the immediate postoperative and recovery periods. They were evaluated by physical therapy and recommended for discharge to home.

The patient was scheduled for follow-up 1 week after discharge; however, the patient missed his follow-up appointment and was subsequently readmitted after an overdose 3.5 weeks after discharge. Follow-up visit was conducted in the emergency department, with a well-healed wound (Figure 2, D). Physical examination at the time of re-presentation included firing tibialis anterior 4/5, extensor hallucis longus 5/5, flexor hallucis longus 4/5, and gastroc-soleus 4/5. Sensation was present to light touch in superficial peroneal, deep peroneal, and tibial distributions, and the patient had a palpable dorsalis pedis pulse. During his visit to the emergency department, the patient refused follow-up radiographs of his fracture. He subsequently left the emergency department against medical advice and was lost to follow-up.

## Discussion

Our case is an example of a high-energy mechanism direct injury to the fibula in a young patient not on anticoagulation resulting in compartment syndrome. Previous reports involving isolated fibula fractures, though sparse in the literature, comprised cases where the patient was anticoagulated.^5-8^ Anticoagulation provides another mental “red flag” when considering whether to trend compartment examinations in patients with injuries not classically associated with compartment syndrome.^2,9,10^ However, when patients have no “red flags” other than injury mechanism, compartment syndrome is more easily missed.^10^

Previous case series have demonstrated that the development of compartment syndrome in the absence of fracture often causes delays in treatment with more muscle death than in cases of acute compartment syndrome associated with fracture.^2,11^ Our case was caught rather early and compartment measurements were conducted promptly, resulting in immediate compartment release in the OR within 3 hours of arrival, while previous series reported an average 34 hours of delay in release.^2,11^ Our case demonstrates the importance of maintaining high suspicion for compartment syndrome based on the mechanism, rather than relying on injury burden alone.^12^ Furthermore, our case demonstrates the importance of early, complete physical examination for all high-energy injuries regardless of injury burden. Prompt, thorough examination allowed for expeditious surgical management in this case, minimizing soft-tissue injury for this patient.

Other cases have been published regarding atypical compartment syndromes. Bartoníček et al discussed a case of missed compartment syndrome after a Bosworth fracture in a 39-year-old man. They describe a patient who underwent multiple attempts at closed reduction of a Bosworth fracture with subsequent development of pain, paresthesia, and swelling, which went unrecognized as a compartment syndrome because of the atypical association with ankle fracture-dislocations. The fracture was fixed definitively 13 days after injury; however, paresthesia and swelling persisted, resulting in a fixed flexion contracture of the great toe and diminished sensation of the toes and first interdigital web space.^13^ Furthermore, Ng et al^14^ discussed an atypical case of nonpainful compartment syndrome where the patient, also an intravenous drug user, presented with an erythematous and swollen forearm, which was presumed to be cellulitis or a vascular issue, delaying the ultimate diagnosis of compartment syndrome. Intravenous drug use can further cloud the clinical picture, as in our case, leading to delayed diagnosis. In another instance of atypical compartment syndrome, Shields et al described a young female patient with foot pain after an extended period of marching, ultimately being diagnosed with compartment syndrome and undergoing foot fasciotomies.^15^ This example of nonstandard, atraumatic compartment syndrome in the foot, an unusual yet described anatomic location for compartment syndrome, reminds us that compartment syndrome may include areas other than the leg or forearm. Other previously described areas of atypical compartment syndrome include the thigh, buttock, hand, and foot. Bearing in mind this broad spectrum of compartment syndrome presentations, including our case, reminds the clinician to frequently consider compartment syndrome on the differential, even in unlikely scenarios.

Obtaining compartment measurements in patients with suspected compartment syndrome can be a useful adjunct to the physical examination, and compartment measurements can be especially useful in atypical presentations such as ours.^16^ Classically, compartment pressure monitoring has been described for obtunded patients where the physical examination is unreliable or incomplete.^11^ However, compartment monitoring may also prove useful early in the evaluation of patients with minimal injury burden, but with high suspicion of impending compartment syndrome and equivocal or unreliable examinations. As it relates to our case, our patient had mild-to-moderate pain with passive extension of the ankle and toes and had normal motor and sensory examinations of the foot. However, clinical suspicion for early compartment syndrome remained high because of the firmness of his leg, especially when compared with the contralateral. Given the unreliable examination, we found it pertinent to use compartment pressures as an adjunct to our clinical examination to confirm our suspicions, which ultimately provided supplemental evidence to indicate early surgical management. It is crucial to understand that diagnosing compartment syndrome using clinical findings alone has an estimated sensitivity of 13% to 54%, when compared with the slit catheter technique (compartment pressure monitoring device) with an estimated sensitivity and specificity of 94% and 98%, respectively.^17^

It has been classically described that when intracompartmental pressures increase to within 30 mm Hg of preoperative diastolic pressures, this indicates inadequate perfusion consistent with compartment syndrome**.**^18^ However, our case demonstrates a challenge to this classic definition because diastolic pressure at the time of compartment measurement was 102 mm Hg. This predicament is common in the assessment of compartment syndrome in acute trauma patients who may be hypertensive for a variety of reasons, including pain, baseline hypertension, and medications given in the field. An alternate, yet important definition of compartment syndrome that has previously been defined is “absolute compartment pressure anywhere from 30 to 50 mm Hg.”^19-21^ This alternate definition may be overlooked by a younger resident or less experienced practitioner, who may have the misconception that high diastolic pressure indicates adequate venous return regardless of absolute elevation of compartment pressures. However, high compartment pressures promote fluid extravasation regardless of elevated diastolic pressure, and thus, absolute intracompartmental pressure increase must be treated with equal seriousness as pressures <30 mm Hg from the diastolic. In this case, using a combination of intracompartmental pressure measurement and clinical judgment was necessary to reinforce suspicion for the diagnosis given an atypical presentation.

## Conclusion

Even in the case of isolated fibula fracture, the mechanism of injury (high-energy trauma, crush, etc) must be considered as a risk factor of the development of compartment syndrome. Atypical presentations of acute leg compartment syndrome are underrepresented in the literature. This case report demonstrates a presentation of acute compartment syndrome in the absence of the usual risk factors, such as tibia fracture or anticoagulation, and in the setting of a vague clinical presentation that required high suspicion and clinical judgment to diagnose. Patients with clinical signs of compartment syndrome such as pain out of proportion on examination should be monitored closely even with isolated fibula fractures when a high-energy mechanism is present. There should be a low threshold for compartment pressure measurement and surgical management if clinical suspicion is high.
